# Sub-cycle ionization dynamics revealed by trajectory resolved, elliptically-driven high-order harmonic generation

**DOI:** 10.1038/srep39006

**Published:** 2016-12-19

**Authors:** E. W. Larsen, S. Carlström, E. Lorek, C. M. Heyl, D. Paleček, K. J. Schafer, A. L’Huillier, D. Zigmantas, J. Mauritsson

**Affiliations:** 1 Department of Physics, Lund University, P.O. Box 118, SE-221 00 Lund, Sweden; 2 Department of Chemical Physics, Lund University, P.O. Box 124, SE-22100 Lund, Sweden; 3 Department of Chemical Physics, Charles University in Prague, Ke Karlovu 3, 121 16 Prague, Czech Republic; 4 Department of Physics and Astronomy, Louisiana State University, Baton Rouge, Louisiana, 70803, United States of America

## Abstract

The sub-cycle dynamics of electrons driven by strong laser fields is central to the emerging field of attosecond science. We demonstrate how the dynamics can be probed through high-order harmonic generation, where different trajectories leading to the same harmonic order are initiated at different times, thereby probing different field strengths. We find large differences between the trajectories with respect to both their sensitivity to driving field ellipticity and resonant enhancement. To accurately describe the ellipticity dependence of the long trajectory harmonics we must include a sub-cycle change of the initial velocity distribution of the electron and its excursion time. The resonant enhancement is observed only for the long trajectory contribution of a particular harmonic when a window resonance in argon, which is off-resonant in the field-free case, is shifted into resonance due to a large dynamic Stark shift.

The process of high-order harmonic generation (HHG)^1^,^2^ driven by a strong infrared (IR) laser field interacting with a rapidly ionizing medium is the main light source for the field of attosecond science^3^,^4^,^5^,^6^. The HHG process can be used to produce attosecond pulses because there is a natural, sub-cycle electron dynamics built into the physics of HHG^7^,^8^, which leads to a very broad plateau of emitted harmonics. This means that studying the HHG process itself in detail can, in principle, provide a deeper understanding of strong field electron dynamics at the attosecond time scale. Over the last decade experiments^9^,^10^ have indeed shown that the sub-cycle dynamics of HHG are encoded in the harmonic spectrum, though extracting them is complicated because of the highly non-linear nature of the process.

Much of the promise in using HHG to better understand strong field physics at the sub-cycle level can be attributed to the effectiveness of the simple, semi-classical three-step model commonly used to describe the generation process^11^,^12^. In this model, an electron is first tunnel ionized and then accelerated by a strong laser field. If the electron is driven back to the vicinity of the ion by the oscillating strong field, the accumulated energy may be emitted as a photon^11^,^12^,^13^ when the electron and ion recollide. The sequence of ionization and return times leading to a specific harmonic frequency is loosely referred to as a trajectory because much of the physics can be understood by considering classical electron trajectories in a strong laser field, ignoring atomic effects after the ionization step and before the return. Depending on when during the laser cycle the ionization occurs, the electron will have different excursion and return times to the ion leading to different photon emission frequencies, resulting in a comb of odd harmonics of the laser field if the process is repeated over many laser cycles. Even in this simple model, however, there is not a one-to-one correspondence between the harmonic emission strengths and specific trajectories, because there are different trajectories leading to the same final energy. Trajectories that lead to the same photon energy interfere at the single atom level. The effect of this can either be studied^14^,^15^,^16^ or circumvented, for example, through phase matching or spatial separation in the far field of the harmonics as is done in the present work.

Studying trajectory resolved contributions to the HHG spectrum is an attractive proposition because different trajectories probe very different ionization conditions and have different excursion times. The most prominent contributions to the harmonic emission strengths come from the so-called short and long trajectories, which have excursion times of less than one laser cycle. Within a laser cycle the long trajectories are ionized close to the peak field strength and have an excursion time exceeding 0.65 laser cycles. The short trajectories are ionized at low field strengths and have shorter excursion times. Fortunately, the emission from these two trajectory classes can be separated experimentally in the far field enabling, for each harmonic frequency, comparison between ionization at two different sub-cycle field strengths, followed by two different excursion times. This requires, ideally, that high accuracy measurements of both long and short trajectory contributions to each harmonic are made in the same experimental setup^17^.

Until recently most experimental efforts that make use of high harmonics have been concentrated on optimizing HHG from short trajectories, since their emission is well collimated and spectrally narrow. In addition, trains of attosecond pulses have been successfully created and measured by selecting the short trajectories’ contributions^3^,^18^. The emission from the long trajectories is more challenging to use because it is spectrally broader and more divergent, hence it is usually removed by spatial filtering and/or the selection of specific phase matching conditions in experiments as they otherwise can affect the temporal structure of the attosecond pulses^19^. In this paper we report on measurements made with very well-controlled, high repetition-rate laser pulses, which allow us to make trajectory resolved HHG measurements in argon gas while varying the ellipticity and the peak field strength of the driving laser pulses. The results allow us to elucidate new features in the sub-cycle ionization step that lead to long trajectories, that is, ionization at high field strengths followed by long excursion times.

In this article, we present two methods of probing sub-cycle strong-field dynamics by comparing the trajectory resolved emission of high harmonics and then studying the long trajectories in depth. In the first part of the article, a detailed experimental comparison of the ellipticity dependence as a function of harmonic order is presented, for both the short and the long trajectories. While harmonic generation using elliptically polarized driving fields has been extensively studied for the short trajectories both experimentally and theoretically^20^,^21^,^22^,^23^,^24^,^25^,^26^, the polarization dependence of the long trajectories have so far only been investigated theoretically^24^,^25^,^27^. It follows from the simple three-step model that harmonic generation will be very sensitive to the ellipticity of the driving laser since the field acting on the electron while it is far from the ion can cause it to miss the recollision. Since the long and the short trajectories have different excursion times, the impact of changing the ellipticity will be different for the two classes of trajectories.

To explain the ellipticity dependence of the short trajectories it is sufficient to include wave packet spreading due to quantum diffusion, which we can model by including a distribution of momenta transverse to the instanteneous field vector at the moment of ionization. This distribution does not need to depend in detail on the moment of ionization, since the ionization field strength is low for short trajectories. In order to explain the ellipticity dependence of the long trajectories, however, this simple quantum diffusion model is not enough. Due to a larger variation in ionization field strength for the different long trajectories, a field-strength dependent momentum distribution has to be taken into account. We expect that at higher field strengths a broader transverse momentum distribution results from the lowering of the ionization barrier. We include this effect in our theoretical analysis of the long trajectory data via a simple extension of the three step model and find that it fits the our experimental data very well over the HHG plateau.

In the second part of our study, the sub-cycle sensitivity of trajectory resolved HHG measurements is used to study a region of the spectrum in which atomic resonances can alter the HHG signal. In particular, a window resonance in argon that is far from any harmonic of the laser frequency in the field-free case is shown to have a large effect on the long trajectory harmonic closest to it, but little or no effect on the short trajectory. We attribute this to the fact that the long trajectory component is dynamically Stark shifted into resonance by the laser field, which leads to a drastic enhancement of the emission from the long trajectory, but not the short where the field strength is much weaker and is not sufficient to shift the state into resonance. We measure this effect for a set of resonant harmonics over a range of driving field intensities.

## Experimental setup

The experimental setup used for the experiment presented in this article is described in a recent publication^28^ and is briefly outlined here. An Yb:KGW based laser system (“Pharos”, Light Conversion Ltd.) was used to deliver 170 fs, pulses with a central wavelength of 1030 nm. The laser system has a variable repetition rate between 1 and 600 kHz, but all the presented data were recorded at a repetition rate of 20 kHz. The pulses were focused tightly into a continuous argon gas jet, with a 90 m orifice, using a 100 mm focal length achromatic lens. Directly after the interaction region, a differential pumping hole with an inner diameter of 0.5 mm was placed to minimize the background gas in the detection chamber. The differential pump hole allowed for a pressure difference of the background gas between the generation and detection chambers of 4–5 orders of magnitude. The HHG spectrum was measured using a home-built imaging spectrometer based on a variable-line-spacing grating and a microchannel-plate with an attached phosphor screen and a camera with a resolution of 2456 × 2058 pixels and a dynamic range of 14 bits. The grating diffracts and refocuses the XUV in the horizontal direction while the vertical direction is left unaffected. Therefore the vertical direction provides the divergence of the XUV light while the horizontal direction shows the spectrum.

## Ellipticity measurement

**Figure 1:**
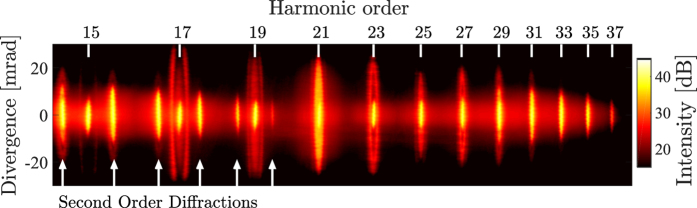
Typical harmonic spectra optimized to generate harmonics from both the short and the long trajectories.

Figure 1 shows a typical harmonic spectrum when a linearly polarized driving laser is used and the gas jet is placed in the focal plane of the generating beam. The experimental parameters (pulse energy, gas density, spot size, etc.) were optimized to generate harmonics from both the short and the long trajectories.

The Gaussian transverse and temporal intensity profile of the driving laser, in combination with the fact that the dipole phase of the long trajectories has a stronger intensity dependence than the short trajectories, result in a larger wavefront curvature and more divergent light generated by the long trajectories^29^,^30^,^31^, this also explains the spatial–spectral rings observed in the far field. We therefore attribute the inner part of the harmonic spectrum to be dominated by the short trajectories, while the spatial–spectral rings are attributed to interference between long trajectories of different emitters. As the trajectory dependent dipole phase is strongest for the low orders, the interference rings are mainly seen for the low end of the plateau region. This spatial separation was exploited to study the contributions from the long trajectories only.

**Figure 2:**
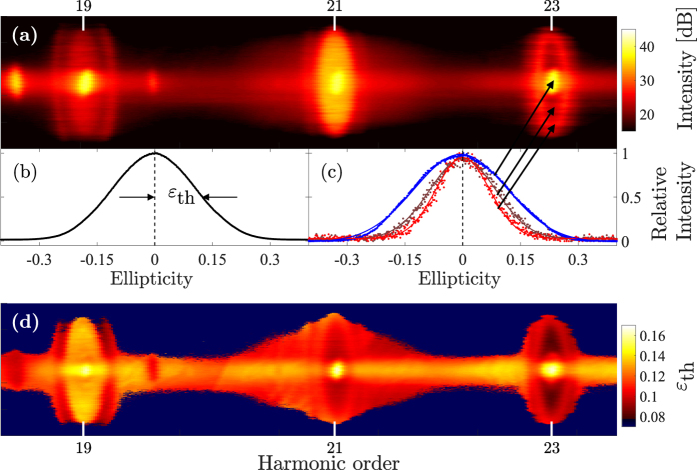
(**a**) Enlarged view of the harmonic spectrum at linear polarization for H19–H23 of Fig. 1. (**b**) Spatially and spectrally integrated signal of H23 as a function of ellipticity. (**c**) Measurement of the ellipticity dependence of three different spatial–spectral parts of H23 as indicated by the three arrows. The solid lines represent Gaussian fits to the experimental data. (**d**) Pixel-by-pixel threshold ellipticity of the spatial–spectral region of part (**a**).

A quarter-wave plate was used to introduce ellipticity, defined as the ratio between the minor and major axis components of the driving laser field. Figure 2(a) shows an enlarged view of the spatial–spectral profile of harmonics 19–23 of Fig. 1. Figure 2(b) shows a measurement of the spatially and spectrally integrated strength of harmonic 23 (H23) as a function of ellipticity of the driving laser relative to the strength at linear polarization. The integrated signal clearly follows a Gaussian distribution with respect to ellipticity as previously observed^20^.

We define the threshold ellipticity, *ε*_th_, as the amount of ellipticity required for the harmonic signal to drop by a factor of two compared with linear polarization. Our very high signal-to-noise ratio allows us to analyze the ellipticity dependence of each pixel rather than the spatially and spectrally integrated signal. Figure 2(c) presents the strength of three different pixels within H23 as a function of ellipticity. The strength of each pixel is fitted with a Gaussian profile to extract the corresponding threshold ellipticity of each pixel, which are used to create a two-dimensional map of the threshold ellipticity as a function of energy and divergence angle. The full threshold ellipticity maps for the conditions of Fig. 1 can be found in the Methods section. Focusing on H23 in Fig. 2(d), we observe three different regions of threshold ellipticity; an inner region with a threshold ellipticity around 0.16, and two outer regions with threshold ellipticities of around 0.09 and 0.1 respectively.

**Figure 3:**
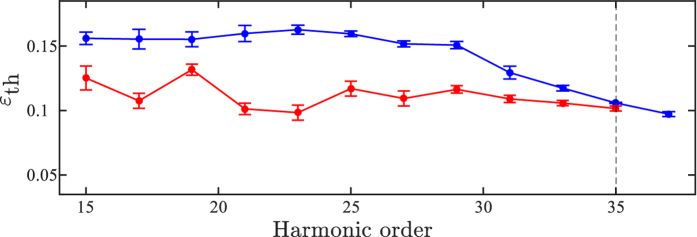
The red (blue) line show the measured threshold ellipticity as a function of harmonic order for the long (short) trajectories when the laser is focused in the middle of the gas jet. The error bars indicate the standard deviations of the corresponding threshold ellipticity.

Figure 3 presents the average threshold ellipticity for both the long and the short trajectories as a function of harmonic order for the conditions of Fig. 1. For the short trajectories, we observe a behavior similar to previous measurements^20^,^22^,^26^, *i.e.*, the threshold ellipticity decreases slowly with increasing harmonic order. A similar trend is also observed for the long trajectories, in contrast to what would be expected if only the excursion time of these trajectories is considered.

## Ellipticity theory

In a semi-classical model where the propagation step is calculated classically and the electron only has a velocity parallel to the electric field, only linearly polarized light would produce high-order harmonics since any ellipticity will prevent the electron from returning to its original position. The fact that high-order harmonics are observed even for elliptically polarized light is usually attributed to quantum diffusion; the electron wave packet spreads out as it is accelerated in the laser field. The wave packet spread allows for an overlap between the electron and the parent ion, even when the electron is transversely displaced due to the elliptically polarized laser field.

Quantum diffusion can be seen as resulting from an initial distribution of velocities of the electron – the more confined the electron is in one direction, the more it will spread. In particular, a spatial confinement in the direction perpendicular to the laser field, will lead to a transverse velocity distribution which is necessary for HHG. A rough estimate of the confinement is given by the size of the groundstate. Using this estimate, a trajectory spending longer time in the continuum will diffuse more which results in a lower HHG yield.

For the short trajectories, the above estimate of the quantum diffusion, which is independent of the ionization time, is sufficient to explain the increase in sensitivity, as a function of harmonic order. For this set of trajectories the highest energy photons are produced by electrons with the longest excursion time. As the transverse displacement of the electron at the point of recombination increases with the excursion time, the trajectories leading to the higher harmonics are displaced more than those leading to the low orders. Therefore the overlap between the ion and the electron at the recombination time decreases with harmonic order.

For the long trajectories, this effect leads to the opposite result, as the kinetic energy of the returning electrons decreases with increasing excursion time. To understand the experimentally observed ellipticity dependence of this set of trajectories, we apply a model that also takes the sub-cycle variation of the initial electron velocity distributions into account, as well as the change in excursion time for the different trajectories as the ellipticity is varied. This effect plays a major role in the initial velocity distribution as the long trajectory electrons are ionized closer to the peak of laser field, where the atomic potential is more distorted in the direction of the laser field, and thus the electron wave packet is more perpendicularly confined at the time of ionization^32^. The perpendicular confinement of the electron at the ionization time leads to a large uncertainty in the perpendicular velocity distribution.

Our method is similar to references 24, 25, 26, but the definition of threshold ellipticity is not the same in the different studies. The procedure is as follows: First, we calculate the return energy of the electrons for the two first sets of trajectories as a function of both ionization time and ellipticity. The position of an electron released at time *t*_*i*_ in an elliptical field 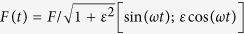 is found by integrating the Newtonian equations of motion twice:


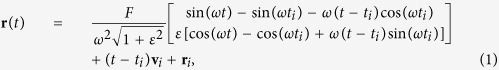


where **v**_*i*_ and **r**_*i*_ are the initial velocity and position, respectively; *F* is the field amplitude, *ω* the frequency of the fundamental field, *ε* ∈ [−1, +1] is the ellipticity, with 0 meaning linear polarization along the *x* axis. Atomic units are used. We assume that **r**_*i*_ = **r**(*t*_*r*_) = 0, where *t*_*r*_ is the moment of return. Finding this time requires solving the transcendental equation numerically. For elliptical polarization, the drift acquired by the electron can be countered by an initial velocity **v**_*i*_ that is transverse to the driving field at the time of ionization (this is analogous to quantum diffusion of the electron wavepacket as it is accelerated in the laser field). Thus, we solve (1) for *t*_*r*_ and **v**_*i*_ for each *t*_*i*_ ∈ [0.25*T*, 0.5*T*], *T* being the period, and each *ε* ∈ [0, 1]. We assume that 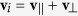, where the two components are parallel and perpendicular to the driving field at the time of ionization. Furthermore, we assume that *v*_||_ = 0, such that all uncertainty is in the initial transverse momentum, *p*_⊥_ = *m*_e_*v*_⊥_, (*m*_e_ = 1 in atomic units).

**Figure 4:**
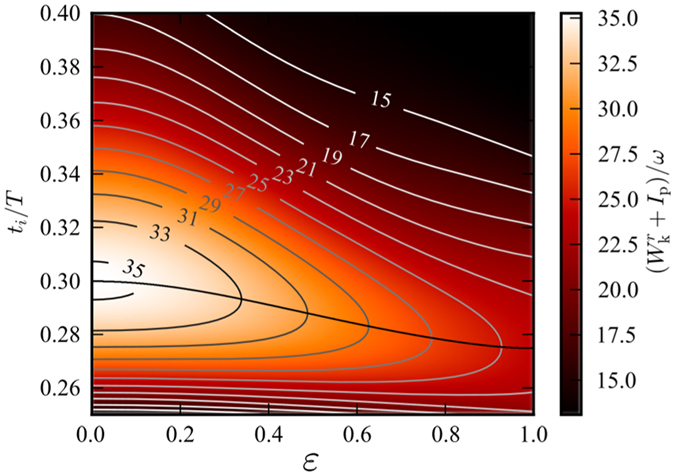
Map of return energies, as a function of ellipticity *ε* and ionization time *t*_*i*_. *I*_p_ is the ionization potential of the ground state. Plotted are also isoenergetic curves corresponding to the harmonics of the fundamental field, and the cut-off energy (shown by the solid black line), which decreases for increasing ellipticity. Trajectories, which are ionized earlier than the cut-off energy, correspond to the long trajectories. It is easy to see that a specific time of ionization does not correspond to a certain recombination energy.

The kinetic energy at the time of return is given by 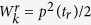; this gives the map of energies seen in Fig. 4.

**Figure 5:**
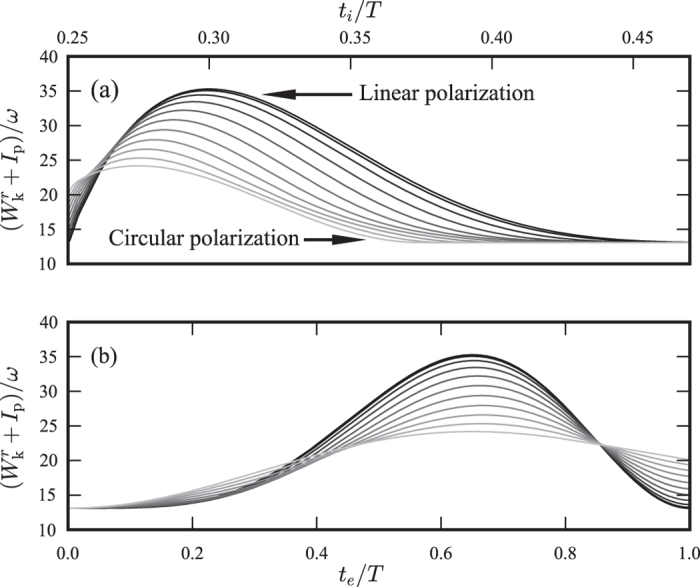
(**a**) Lineouts of Fig. 4 for 11 equidistant ellipticities from linear to circular polarization. From this plot it is obvious that the cut-off decreases with increasing ellipticity and that it occurs for earlier ionization times. It is also clear that the initial timing necessary to produce a specific harmonic changes with the ellipticity. (**b**) The same as (**a**), but plotted as a function of excursion time *t*_*e*_ = *t*_*r*_ − *t*_*i*_ instead.

As can be seen in Fig. 4, the cut-off position is shifted when the ellipticity is increased (*i.e.* the highest energy photons can only be produced from linearly polarized light) and the initial timing leading to a specific harmonic order is also changed. This trend is even more clear when lineouts at different ellipticities are presented as in Fig. 5.

The next step to estimate the harmonic yield is to calculate the combined probability of ionizing at time *t*_*i*_ and having the required initial velocity for the electron to return. This is possible since the correspondence between a certain harmonic and its ionization time for different ellipticities is already calculated. Since the required transverse momenta for the long trajectories to return are quite large for high ellipticities, we use the full expression for the transverse momentum distribution found in reference 33,


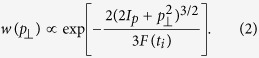


**Figure 6:**
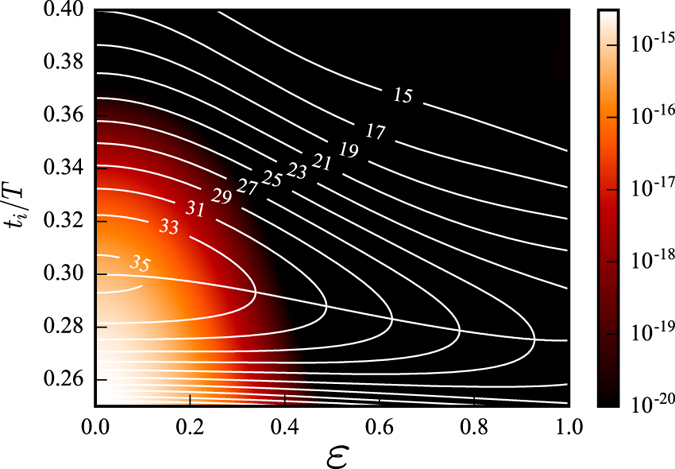
Map representing the combined probability of ionizing at time *t*_*i*_ and having the initial transverse velocity required for return as a function of ellipticity and ionization time. The isocurves are the same as in Fig. 4, representing constant return energy. Following an isocurve gives the probability of generating a certain harmonic, as a function of ellipticity.

*I*_p_ is the ionization potential of the ground state. The tunneling rate is taken from ADK theory^34^. This combined probability, which is the product of the separate probabilities described above, is displayed using a colour scale in Fig. 6, as a function of ellipticity and ionization time.

The isoenergetic curves from Fig. 4 are also included in the figure. To calculate the yield of a given harmonic order as a function of ellipticity, one extracts the probability along the corresponding isoenergetic curve.

**Figure 7:**
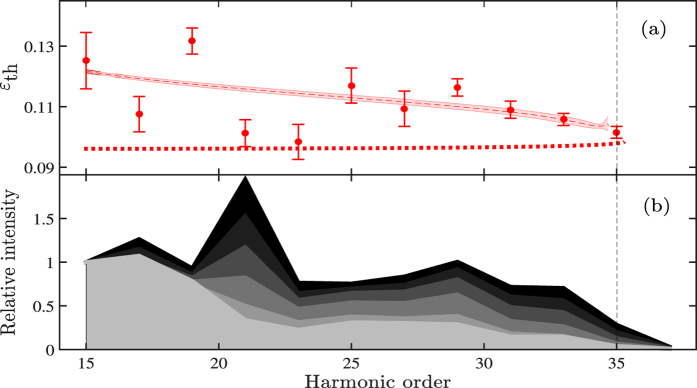
(**a**) The dashed lines show the numerically calculated threshold ellipticities for the long trajectories in the plateau region according to our model. Correspondingly, the dotted lines show the threshold ellipticity given by ref. 25. For comparison, the experimental data for the long trajectories are shown. The simulations were done for a laser intensity of 8.5 ⋅ 10^13^ W/cm^2^ and a wavelength of 1030 nm. (**b**) Integrated harmonic spectra for in-focus generation with various ellipticities. The color scale corresponds to different ellipticities from black (*ε* = 0) to light gray (*ε* = 0.25) in steps of 0.05. The fillings between the different harmonics allow us to better visualize the differences. The various spectra have been normalized to the level of H15. The error bars used in (**a**) show the standard deviations of the threshold ellipticities of the corresponding harmonic.

Finally, in the last step of our model, the calculated yield as a function of ellipticity is fitted with Gaussian functions for each harmonic to obtain the threshold ellipticities in a similar manner to the experimental data. The result of the model for the long trajectories is presented in Fig. 7(a) together with the experimental data.

Our extended model compares very well with the experiment presented in this work; in particular the decrease in threshold ellipticity with increasing harmonic order is explained. This is opposite to what would be expected from sub-cycle field-independent quantum diffusion. It is also opposite to the analytical expression presented in reference 39 which is included for comparison in Fig. 7(a) as a dotted line.

From Fig. 7(a) it is clear that some of the long trajectory harmonics (17, 21 and 23) have a lower threshold ellipticity than what is predicted by the model. We attribute this to the presence of atomic resonances in the vicinity of the corresponding energies, which clearly cannot be captured by the model we are using. In what follows, we demonstrate that these resonances can be dynamically Stark shifted by the sub-cycle field strength and will therefore influence the harmonic generation differently for the short and the long trajectories. The spatial separation of the short and long trajectories leading to the same energy, enable us to directly compare the influence of the sub-cycle field strength. For a given harmonic order, the short trajectory is initiated at a field strength which is insufficient to shift the state into resonance, thereby precluding the enhancement observed for the long trajectory initiated at a higher sub-cycle field strength.

## Resonant HHG

**Figure 8:**
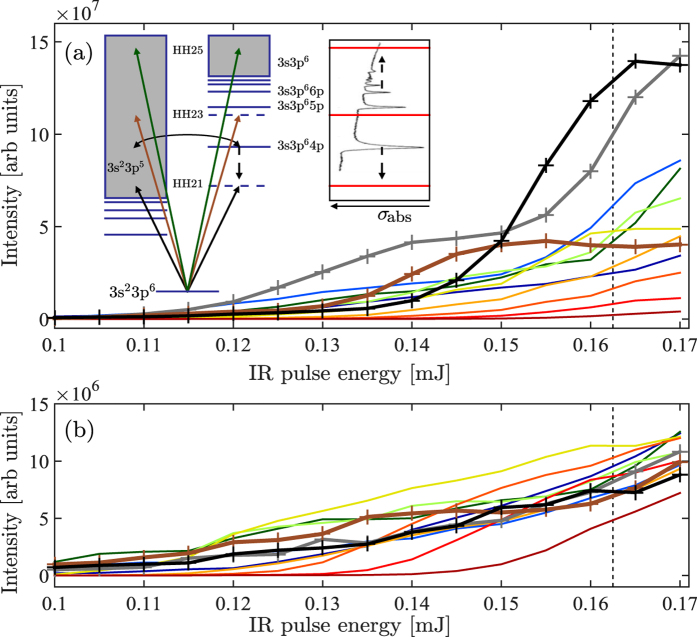
Experimentally measured long (**a**) and short (**b**) trajectory intensities of H15–H37 as a function of pulse energy of the fundamental field. H17 is shown in gray, H21 in black, and H23 in brown, while the remaining harmonics are shown in a rainbow color scale from dark blue (H15) to dark red (H37) with increasing order. The dashed line indicates roughly the conditions of Fig. 1. The inset of (**a**) schematically shows how the Rydberg series for the 3s electrons in combination with the continuum for the 3p electrons creates a series of window resonances. Absorption of an XUV photon can create a coherent superposition of the two valence electrons which interfere and affects the absorption cross-section *σ*_abs_^42^. In argon this amounts to a reduction of the absorption cross-section^43^. The vertical arrows within the inset indicate the expected direction of the light induced energy-shift of the respective 3s^1^3p^6^np^1^ states.

Resonant HHG in argon at a photon energy corresponding to H17 in our experiment has previously been observed^35^. In our study, we also see an effect for H21 and H23 [Fig. 7(b)]. We will focus the discussion on H21, where the effect is most pronounced. At linear polarization a strong enhancement of H21 is clearly observed, while this enhancement is gone for an ellipticity of 0.2 as can be seen in Fig. 7(b). The change in ellipticity leads to a variation in the intensity, since the pulse energy is kept constant. This means that the observed effect can be due to either the intensity or the ellipticity. In order to disentangle the two, an intensity scan was performed for linearly polarized light. Figure 8 shows the experimentally measured intensities of the long (a) and short (b) trajectories for H15–H37 as a function of IR pulse energy. As clearly observed, the yield of H21 rises more rapidly for the long trajectories once the IR pulse energy exceeds 0.14 mJ [Fig. 8(a)], while the emission from the short trajectories are left unaffected [Fig. 8(b)]. Enhancement of the long trajectories can also be clearly seen for H17 and H23, albeit, at slightly lower pulse energies. Harmonic 17 qualitatively follows the trend predicted for the single atom response given in reference 35, thus H17 is not further discussed in the present work. Harmonic 23 reaches a maximal strength at 0.15 mJ whereafter a slow decrease with respect to increased pulse energy is observed.

We interpret the behavior of H21 (but also H17 & H23) to be the result of HHG in the presence of an atomic resonance. Resonant HHG may increase the harmonic yield through a number of different mechanisms^35^,^36^,^37^,^38^,^39^,^40^,^41^. For H21 with photon energy of 25.2 eV, the closest resonance is the 3s^2^3p^6^ → 3s^1^3p^6^4p^1^ transition (26.6 eV), which is a window resonance^42^,^43^. Our interpretation requires that the 3s^1^3p^6^4p^1^ state, which is lowest state in the 3s → np closed channels, is red-shifted by approximately 1.4 eV [see inset in Fig. 8(a)]. This is feasible as the dynamical Stark shift of the 3s^1^3p^6^4p^1^ state should be dominated by the interaction with the 3s → np closed channel, rather than through coupling with the 3p → Σ(s, d) open channels^44^,^45^, and the Stark shifts on the order of the ponderomotive energy are well known^46^,^47^. In addition to the Stark effect, the IR intensity also causes a blueshift of the IR energy, and thereby the XUV photon energies. However, it was confirmed from the intensity scan that this effect is too small to explain the results, as the central frequencies of the harmonics did not change. Since we only observe the enhancement for the long trajectory, we interpret this as an effect of the comparably higher field strength for this trajectory, at the time of ionization.

Reshaping of the argon HHG spectrum by this particular window resonance has previously been observed^48^, however, the interpretation is fundamentally different in the work presented here. In reference 48, a few-cycle pulse was used to generate broadband harmonics by HHG in a gas jet. The backing pressure for the continuous gas jet was then increased significantly so that all other wavelengths than exactly the resonant wavelength were suppressed by re-absorption in the generating gas. This led to a narrowing of H17 (of 800 nm) from a width of roughly 1.5 eV to a width comparable to the field-free width of the window resonance^43^. This leads us to believe that the effect observed in reference 48 happens over a large volume, where the IR intensity is weak. The results presented in this article, however, is clearly an effect that take place at high laser intensity, where the dynamical Stark effect is strong.

The behavior of H23 with respect pulse energy of the driving laser can be understood as an effect of over-shifting of the atomic resonances causing the enhancement. As indicated in the inset of Fig. 8(a) the field-free detuning of H23 is less than the detuning of H21 with respect to both the transition energies into the 3s^1^3p^6^4p^1^ and the 3s^1^3p^6^5p^1^ states, so the minimal required pulse energy for enhancement of this harmonic is lower. Nevertheless, the maximal enhancement factor is largest for H21, due to the strong dipole coupling with the red-shifted 3s^1^3p^6^4p^1^ state.

As the pulse energy is increased beyond the optimum energy for resonant generation of H23 the enhancement of this harmonic starts to vanish. The slow decrease likely originates from the long pulse duration of the driving laser, which means that a number of cycles will have the optimum energy shift. A similar effect is expected to occur for H21 at higher pulse energies, however, due to limitations of the laser system this was not seen in the present work.

Apart from the major effects on H17, H21 and H23 observed both in the ellipticity and the intensity measurements a minor amount of enhancement of H19, H25 and H27 can be observed for the long trajectories once the IR pulse energy exceeds 0.15 mJ [Fig. 8(a)]. The field-free detuning from the 3s3p6np manifold of resonances are larger for these harmonics, so any enhancement effect on these harmonics is both expected to be less, and to occur at higher pulse energies in full agreement with the observation.

In conclusion, we have experimentally investigated the ellipticity and intensity dependencies of HHG from the long and the short trajectories. This type of measurements enables us to probe the influence of the sub-cycle field strength on HHG process. We have shown that the well-established semi-classical model has to be extended by taking the instantaneous field strength into account, to also describe the general behavior of the long trajectories. We have demonstrated how off-resonant states embedded in the continuum can enhance long trajectory harmonics by being shifted into resonance by the strong driving laser, different amounts for different trajectories due to the sub-cycle nature of the generation process. When the driving laser field is strong enough to cause an enhancement at linear polarization, these harmonics show a stronger ellipticity dependence as the dynamical Stark shift depends on the polarization.

This study highlights the importance of systematical studies of the generation process under various conditions. Furthermore, the extension of the knowledge of the harmonic generation process to the long trajectories will be beneficial for high-order harmonic spectroscopy studies.

## Methods

### Evaluation of experimental data

In this section we present the details of the analysis method used for the experimental data.

It is well-known from the strong field approximation that for harmonics in the plateau region, there are several electronic trajectories, which may contribute to the generation process. Emission from these different trajectories interferes and shapes the far field spatial spectral profile. The phase of these trajectories can be approximated with a phase proportional to the intensity *I(x, y, z, t*) such that


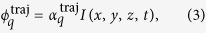


where 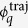 is the trajectory dependent dipole phase and 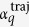 is the proportionality constant. The first two sets of electron trajectories are usually referred to as the short and long trajectories. It well-established that in the plateau region the proportionality constants are much larger for the long trajectories than for the short trajectories^31^,^49^.

**Figure 9:**
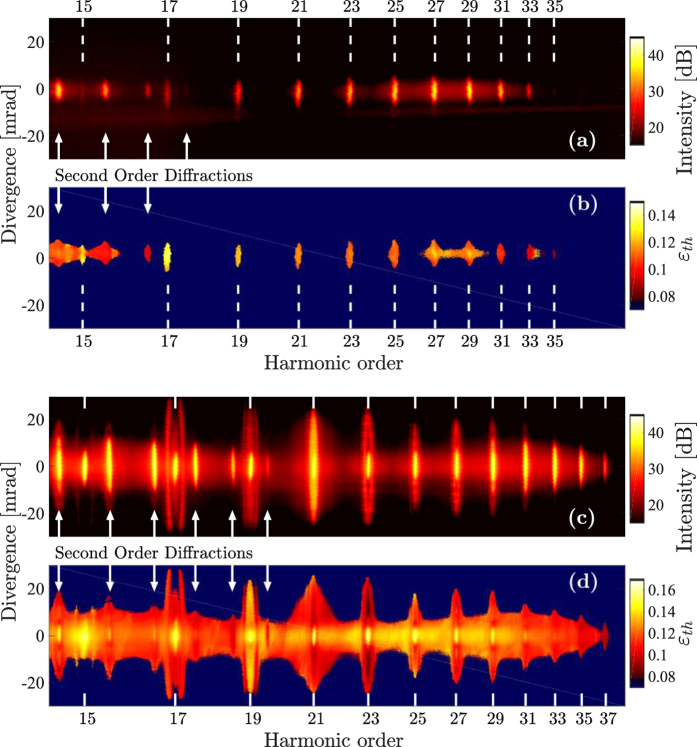
(**a**) Harmonic spectrum at linear polarization when the gas jet is placed behind the focal plane of the laser. (**b**) Threshold ellipticity map as a function of energy and divergence angle for out-of-focus generation. (**c**) Harmonic spectrum at linear polarization when the gas jet is placed in the focal plane of the laser. (**d**) Threshold ellipticity map as a function of energy and divergence angle for in-focus generation.

The short trajectories can be isolated in the generation process by placing the gas jet behind the focal plane and adjusting gas pressure and pulse energy accordingly^50^,^51^. A spectrum optimized for this is shown in Fig. 9(a). Figure 9(b) shows the corresponding threshold ellipticity map, which is extracted in a similar manner as in the main article. When the gas jet instead is placed at the focus of the laser both sets of trajectories can efficiently be phase-matched by adjusting the other experimental parameters accordingly. Figure 9(c) shows a spectrum optimized to generate with both sets of trajectories, while Fig. 9(d) is the corresponding threshold ellipticity map. As a consequence of the larger dipole phase of the long trajectories, the light produced by the these trajectories are more divergent. This effect was used to spatially separate the contributions from only the long trajectories in Fig. 9(c,d).

**Figure 10:**
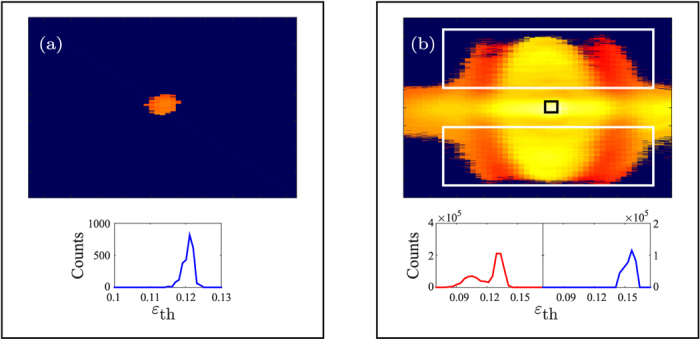
(**a**) An enlarged view of the ellipticity map of H19 for out-of-focus generation [Fig. 9(b)]. The lower panel shows a histogram of the threshold ellipticities. Each pixel is weighted with the corresponding pixel strength at linear polarization. (**b**) An enlarged view of the ellipticity map of H19 for in-focus generation [Fig. 9(d)]. The lower panels show histograms of the threshold ellipticities within the white boxes (left panel) and the black box (right panel). The histograms are weighted with the corresponding pixel strength at linear polarization.

Figure 10 shows enlarged views of H19 for out-of-focus generation [(a)] and in-focus generation [(b)]. In the out-of-focus case the harmonic exhibits a homogeneous spatial-spectral dependence with respect to ellipticity, this is not observed for the in-focus case, where several regions of ellipticity dependence are clearly observed.

The homogeneity of the ellipticity dependence for out-of-focus generation [Fig. 10(a)] reveal that in order to study ellipticity dependence of the short trajectories out-of-focus an imaging spectrometer is not needed and a spatial-spectral integration with respect to harmonic order would be sufficient. This is clearly not the case of in-focus generation [Fig. 10(b)].

**Figure 11:**
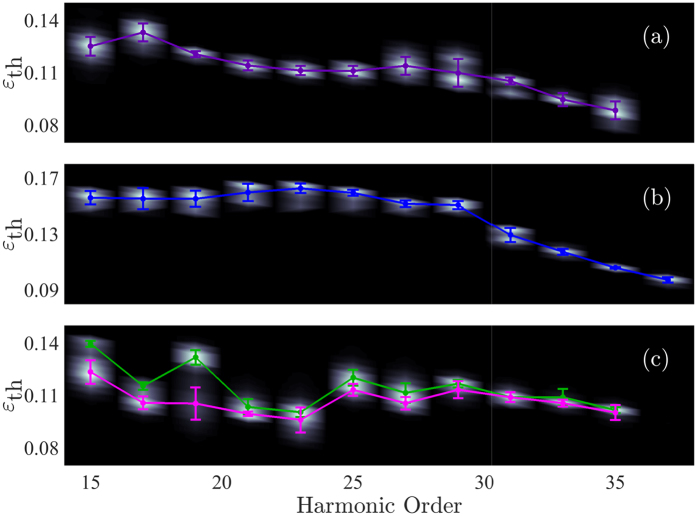
Normalized threshold ellipticity histograms as a function of harmonic order together with the extracted expectation values and standard deviations for: (**a**). The center of H15–H33 for out-of-focus generation. (**b**) The center of H15–H37 for the in-focus generation. (**c**) The outer regions of harmonic H15–H35 for in-focus generation.

Figure 11 shows the threshold ellipticity as function of harmonic order for the conditions of Fig. 9. Figure 11 shows normalized threshold ellipticity histograms as function of harmonic order. In the in-focus generation case both the on-axis emission and off-axis emission are shown, in (b) and (c) respectively, while for out-of-focus case only the on-axis emission is shown in (a). We note that for harmonics close to the cut-off indications of the long trajectory appears also for the out-of-focus case. In order to extract expectation values and standard deviations for the various threshold ellipticities as a function of harmonic order and trajectories the experimental data is smoothened using the Kernel density estimation method^52^,^53^. After smoothing, the data was fitted with two Gaussian distributions for the long trajectories, while the short trajectories where fitted with a single Gaussian distribution. The expectation value of the fitted Gaussian distributions are plotted as solid lines in Fig. 11, while the uncertainty bars show the corresponding standard deviations of the fits.

### Detection efficiency

We measured the ratio in detection efficiency between horizontal and vertical polarization to be 1.38. 3 Three dimensional time-dependent Schrödinger equation calculations^25^ show that the plateau harmonics exhibit a smaller ellipticity than the driving infrared laser. We therefore estimate that the upper limit of the non-fixed major axis impact on the measurement to be given by the following expression:


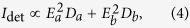


where *E*_*a*_ and *E*_*b*_ are the major and minor axis component of the infrared electrical fields, and *D*_*a*_ and *D*_*b*_ are the respective detection efficiencies. Using this expression together with the standard Jones matrix calculus for polarization of the infrared light we estimated the upper limit on the determination of the threshold ellipticity to be less than the presented standard deviations. The presented data was performed around the linear polarization direction with the highest detection efficiency. Therefore the threshold ellipticity might be systematically underestimated slightly.

## Additional Information

**How to cite this article**: Larsen, E. W. *et al*. Sub-cycle ionization dynamics revealed by trajectory resolved, elliptically-driven high-order harmonic generation. *Sci. Rep.* **6**, 39006; doi: 10.1038/srep39006 (2016).

**Publisher's note:** Springer Nature remains neutral with regard to jurisdictional claims in published maps and institutional affiliations.
